# Effects of Antimicrobial Peptide Microcin C7 on Growth Performance, Immune and Intestinal Barrier Functions, and Cecal Microbiota of Broilers

**DOI:** 10.3389/fvets.2021.813629

**Published:** 2022-01-07

**Authors:** Ziqi Dai, Lijun Shang, Fengming Wang, Xiangfang Zeng, Haitao Yu, Lu Liu, Jianchuan Zhou, Shiyan Qiao

**Affiliations:** ^1^State Key Laboratory of Animal Nutrition, Ministry of Agriculture Feed Industry Centre, China Agricultural University, Beijing, China; ^2^Beijing Bio-Feed Additives Key Laboratory, Beijing, China; ^3^Fengguangde Laboratory of Sichuan Tieqilishi Group, Mianyang, China; ^4^Department of Immunology, Beijing Key Laboratory of Tumor Systems Biology, School of Basic Medical Sciences, Institute of Systems Biomedicine, Peking University Health Science Center, Beijing, China

**Keywords:** antimicrobial peptides, Microcin C7, broilers, performance, immune function, intestinal health

## Abstract

Microcin C7 is an antimicrobial peptide produced by *Escherichia coli*, composed of a heptapeptide with a modified adenosine monophosphate. This study was performed to evaluate the effects of Microcin C7 as a potential substrate to traditional antibiotics on growth performance, immune functions, intestinal barrier, and cecal microbiota of broilers. In the current study, 300 healthy Arbor Acres broiler chicks were randomly assigned to one of five treatments including a corn–soybean basal diet and basal diet supplemented with antibiotic or 2, 4, and 6 mg/kg Microcin C7. Results showed that Microcin C7 significantly decreased the F/G ratio of broilers; significantly increased the levels of serum cytokine IL-10, immunoglobulins IgG and IgM, and ileal sIgA secretion; significantly decreased the level of serum cytokine TNF-α. Microcin C7 significantly increased villus height and V/C ratio and significantly decreased crypt depth in small intestine of broilers. Microcin C7 significantly increased gene expression of tight junction protein Occludin and ZO-1 and significantly decreased gene expression of pro-inflammatory and chemokine TNF-α, IL-8, IFN-γ, Toll-like receptors TLR2 and TLR4, and downstream molecular MyD88 in the jejunum of broilers. Microcin C7 significantly increased the number of *Lactobacillus* and decreased the number of total bacteria and *Escherichia coli* in the cecum of broilers. Microcin C7 also significantly increased short-chain fatty acid (SCFA) and lactic acid levels in the ileum and cecum of broilers. In conclusion, diet supplemented with Microcin C7 significantly improved growth performance, strengthened immune functions, enhanced intestinal barrier, and regulated cecal microbiota of broilers. Therefore, the antimicrobial peptide Microcin C7 may have the potential to be an ideal alternative to antibiotic.

## Introduction

Antibiotics are used widely in livestock production as an effective antimicrobial drug because they play important roles in disease treatment, disease prevention, and growth promotion (1, 2). Global consumption of antimicrobials in livestock production was approximately 131,109 tons in 2013 and is projected to be 200,235 tons by 2030 (3). Antibiotic use brings enormous economic benefit, but excessive use can lead to development of antimicrobial resistance and drug residues in animal products consumed by humans (1, 4). Antimicrobial resistance makes treatment of bacterial infections with antibiotics less effective and creates a significant threat to the health of livestock and humans (5, 6). Moreover, the number of newly developed and approved antibiotics is decreasing dramatically (7). Therefore, it is of great urgency to find novel alternatives for antibiotics in livestock production.

AMPs are small cationic molecules with amphipathic structures, widely distributed in living organisms (8, 9). AMPs have a broad-spectrum antimicrobial activity against bacteria, fungi, viruses, and protozoa (9). Different from traditional antibiotics, AMPs kill pathogens by lysing cytomembranes or damaging critical intracellular targets (10, 11), which makes it difficult for pathogens to develop antimicrobial resistance (12). In addition to direct bactericidal activity, AMPs are important constituents of the innate immune system and serve as immune regulators. As part of the immune system, AMPs express chemotactic activities, modulate TLR-dependent inflammatory responses, and promote wound healing (9, 13). AMPs secreted by Paneth cells are of great importance in the maintenance of host intestinal health by limiting pathogen colonization and shaping the composition of indigenous microbial communities (14). The impairment of intestinal epithelial barrier and intestinal microecology caused by Paneth cell dysfunction is one of the important pathogenies of inflammatory bowel diseases, such as Crohn's disease and colitis (15). AMPs have important application value in the poultry industry and were reported to have beneficial effect on growth performance, nutrient digestibility, immune functions, and intestinal morphology, integrity, and microflora in broilers (16–18). Hence, AMPs can be potential antibiotic alternatives, which have attracted researchers' interests.

Microcin C7 is a ribosome synthetic heptapeptide with a modified adenosine monophosphate covalently attached to the C-terminus (19), produced by *Escherichia coli* cells harboring a plasmid-borne mccABCDE (20). Microcin C7 acts as a ‘Trojan horse’ antimicrobial peptide and exerts its antimicrobial activity by specifically targeting aspartyl-adenylate, which inhibits protein synthesis (21). Nanomolar concentrations of Microcin C7 exhibit antimicrobial activity against gram-negative strains phylogenetically similar to *E. coli*, including *Klebsiella, Salmonella, Shigella*, and one strain of *Proteus* (22). The features of high safety, stability, and strong antimicrobial ability make Microcin C7 a practical antimicrobial agent that can be applied in the poultry industry, but little is known about its efficacy at present. Hence, the purpose of this study was to determine the effect of dietary supplementation of Microcin C7 on growth performance, immune and intestinal barrier functions, and cecal microbiota of broilers.

## Materials and Methods

### Broiler Management and Experimental Design

In this study, chicks were reared on net-floor cages in closed and ventilated environment. Ten chicks were reared per pen (100 cm × 100 cm × 30 cm) with a separate feeding trough and nipple drinkers. A 24 h constant-lighting program was used. Broilers had *ad libitum* access to feed and water. Room temperature in the first week was controlled between 30 and 34°C, lowered by 3°C each week until a constant temperature of 22°C. Relative humidity was controlled between 50 and 60%. All chicks were inoculated with ND-IB combined vaccine on the 7th day, infectious bursa vaccine on the 14th day, chicken pox vaccine on the 21st day, and Newcastle disease vaccine on the 35th day. The experiment was carried out in the Fengguangde experimental base of the TIEQILISHI Group, Mianyang, Sichuan Province. The animal care protocol was approved by the China Agricultural University Animal Care and Use Committee (No. AW03901202-1-2), and the experiment is conducted in strict accordance to the animal care protocol. The basal diet (Table 1) was antibiotic free and formulated to meet nutritional requirements of broiler chickens recommended by NRC (2012). Coccidiostats were included in the diet.

**Table 1:** Ingredient composition and nutrient concentration of the basal diet (% as-fed)^1^.

**Item**	**Starter phase (1–21 days)**	**Finisher phase (22–42 days)**
**Ingredient**		
Corn (7.8% crude protein)	54.50	55.76
Soybean meal (43% crude protein)	25.00	18.50
Corn gluten meal (56% crude protein)	5.00	5.50
Cottonseed meal (46% crude protein)	4.00	4.00
Flour (ASH <1.5%)	3.00	6.00
DDGS (26% crude protein)	2.50	2.00
Soybean oil	1.70	4.20
Limestone	1.27	1.27
Dicalcium phosphate	1.24	0.87
l-Lysine sulfate (70%)	0.70	0.87
Salt	0.26	0.24
Bentonite	0.20	0.20
dl-methionine	0.18	0.16
DKJ01^2^	0.15	0.13
l-threonine (98.5%)	0.11	0.15
Choline chloride (60%)	0.10	0.08
Hainanmycin (1%)	0.05	0.03
Broiler multivitamin^3^	0.03	0.03
Thermostable phytase (10,000 IU)	0.01	0.01
Total	100.00	100.00
**Chemical composition, calculated (%)**		
Metabolizable energy (kcal/kg)	2,870	3,078
Crude protein	21.47	19.48
Ether extract	4.67	7.12
Crude fiber	3.22	2.82
Crude ash	5.58	4.8
Calcium	0.88	0.78
Total phosphorus	0.67	0.59
Salt	0.32	0.3
Lysine	1.3	1.25
Methionine	0.5	0.45
Cysteine	0.32	0.29
Methionine + cysteine	0.82	0.74
Tryptophan	0.22	0.19
Threonine	0.85	0.80
Arginine	1.36	1.17
Valine	0.94	0.83
Avian digestible lysine	1.19	1.15
Avian digestible methionine	0.47	0.43
Avian digestible cysteine	0.28	0.26

1 *The basal diet for each treatment is the same. The antibiotic control diet included 200 mg/kg 15% methylene salicylate bacitracin and 300 mg/kg 15% aureomycin. The antimicrobial peptide diet included 200, 400, or 600 mg/kg 1% Microcin C7*.

2 *DKJ01 is the trace element premix for chicken. It provided the following per kg of the starter phase feed: Cu (from feed-grade copper sulfate), 12.08 mg; Fe (from feed-grade ferrous sulfate), 77.99 mg; Zn (from feed-grade zinc sulfate), 70.38 mg; Mn (from feed-grade manganese sulfate), 101.74 mg; Se (from feed-grade sodium selenite), 0.3 mg; I (from calcium iodate), 0.5 mg. It provided the following per kg of the finisher phase feed: Cu (from feed-grade copper sulfate), 10.47 mg; Fe (from feed-grade ferrous sulfate), 67.59 mg; Zn (from feed-grade zinc sulfate), 61.00 mg; Mn (from feed-grade manganese sulfate), 88.18 mg; Se (from feed-grade sodium selenite), 0.26 mg; I (from calcium iodate), 0.44 mg*.

3 *Broiler vitamin provided the following per kg of the starter phase feed and the finisher phase feed: Vitamin A, 15,000 IU; Vitamin B_1_, 2.35 mg; Vitamin B_2_, 7.8 mg; Vitamin B_6_, 5.29 mg; Vitamin B_12_, 0.024 mg; Vitamin D_3_, 3,600 IU; Vitamin E, 22.5 mg; Vitamin K_3_, 9.85 mg; Pantothenic acid, 18.71 mg; Nicotinic acid, 89.1 mg*.

One-day-old healthy Arbor Acres broiler chickens (*n* = 300; initial body weight (BW) = 46.67 ± 0.29 g) were obtained from Chengdu Xinjin Yunda poultry breeding cooperative. All chicks were individually weighed and randomly assigned to 1 of 5 treatments with 6 replicates of 10 broilers. Experimental treatments included a corn–soybean basal diet with no additions, a basal diet supplemented with 45 mg/kg chlortetracycline and 30 mg/kg bacitracin methylene disalicylate, and a basal diet supplemented with 2, 4, or 6 mg/kg Microcin C7. Microcin C7, which was isolated from the metabolites of *Lactobacillus johnsonii* by microbial fermentation, was supplied by Angeli (Chongqing) Biotechnology, Co. Ltd (Chongqing, China). The concentration of dietary Microcin C7 was determined based on findings of our previous studies (not published).

### Experimental Procedure and Sampling

The feeding period was divided into two stages: the starter stage (Day 1–Day 21) and the finisher stage (Day 22–Day 42). Broilers were weighed individually at the start of the trial and the end of each phase prior to the morning feeding. Feed consumption was measured at the end of each phase. All feed remaining in the feeder was weighed and subtracted from the daily allowance to determine the actual daily feed intake. The ADG, ADFI, and F/G ratio were calculated. After weighing, two broilers per pen were randomly selected, euthanized, and sampled. Blood was collected under wings, then let stand for 10 min, and centrifuged at 3,000 rpm for 15 min at 4°C, and the supernatant was collected and stored at −20°C for subsequent serum biochemical parameter measurements. After exsanguination from the jugular vein, feathers were removed, and the abdominal cavity was opened. Samples (2 cm) of the duodenum, jejunum, and ileum were excised and washed in PBS and immediately fixed in 4% (v/v) paraformaldehyde solution for characterization of small intestinal morphology. The jejunal and ileal mucosa (about 0.2 g) were scraped, and scrapings were placed in a sterile 2 ml cryopreservation tube, frozen rapidly with liquid nitrogen, and then stored at −80°C for gene expression and total sIgA analysis. Contents of jejunum, ileum, and cecum were removed and placed in a sterile 2 ml cryopreservation tube, frozen rapidly with liquid nitrogen, and then stored at −80°C for microflora measurement.

### Determination of Immunoglobulin and Cytokine in Serum

Serum samples were thawed and thoroughly mixed immediately before testing. Concentrations of serum immunoglobulins A, G, and M and cytokines IFN-γ, IL-1β, IL-10, and TNF-α were measured using commercially available avian ELISA kits (Nanjing Jiancheng Bioengineering Institute, Nanjing, Jiangsu, China), according to standard procedures described by the manufacturer.

### Small Intestinal Morphology

Embedded tissue was deparaffinized and hydrated, cut into slices (5 μm), and then stained with hematoxylin and eosin for morphology measurements as described by Wang et al. (23). Villus height was measured from the tip of the villus to the crypt–villus junction. Crypt depth was defined as the depth of the invagination between adjacent villi (24). All morphological measurements (villus height and crypt depth) were measured on the stained sections under a microscope at ×40 combined magnification (Nikon Eclipse Ci-E, Japan). At least 15 intact, well-oriented crypt–villus units were measured in triplicate per broiler for each intestinal section. Reported values are means from 15 crypt–villus units.

### Quantitative Real-Time PCR for Gene Expression Analysis

Total RNA was isolated from frozen jejunal mucosal samples (50 mg) according to the instructions of the RNAiso Reagent (TaKaRa Bio Inc., Beijing, China). Purity and concentration of total mRNA in samples were evaluated using a spectrophotometer (NanoDrop-2000, Thermo Fisher Scientific, Waltham, MA) at 260 and 280 nm, respectively. Ratios of absorption (260:280 nm) between 1.8 and 2.0 for all samples were accepted as “pure” for RNA. RNA (1 μg) was used to generate cDNA using the M5 Super plus qPCR RT kit with a gDNA remover according to the manufacturer's instructions (MF166-plus-01, Mei5bio, Beijing). Primer sequences for tight junction proteins including ZO-1, Occludin, Claudin 3, Jam-2, mucoprotein Mucin 2, cytokine IFN-γ, TNF-α, IL-8, pattern recognition receptors TLR2 and TLR4, and key linker molecular MyD88 in jejunal mucosa were designed using the GenBank database from the National Center for Biotechnology Information (NCBI) and primer design software. Quantitative real-time PCR was performed with HiPer SYBR Premix EsTaq with Tli RNase H (MF787-01, Mei5bio, Beijing) using a StepOnePlus real-time PCR system (Applied Biosystems) on 96-well plates with 10 μl of total reaction volume of 5 μl HiPer SYBR Premix, 4 μl cDNA, 0.3 μl of forward and 0.3 μl of reverse primers (10 nmol), and 0.4 μl double-distilled water. Each reaction was run in duplicate. The PCR cycling protocol included one cycle of pre-incubation at 95°C for 30 s; 40 cycles of denaturation at 95°C for 5 s and annealing at 60°C for 30 s; one cycle of melting at 95°C for 5 s, 60°C for 60 s, and 95°C for 5 s; and one cycle of cooling at 50°C for 30 s. GAPDH was used as an internal control in this study. Average expression of the target genes relative to GAPDH was determined using the 2^−ΔΔCt^ method as described by Livak and Schmittgen (25). Primers for qRT-PCR were synthesized by Sangon Biotech (Shanghai, China; Table 2).

**Table 2:** The primer sequences amplifying target genes and housekeeping genes.

**Item**	**Forward sequence (5^**′**^ to 3^**′**^)**	**Reverse sequence (5^**′**^ to 3^**′**^)**	**Annealing temperature (**°**C)**	**Product size (bp)**	**Accession number**
*GADPH*	CTGTTGTTGACCTGACCTGC	TCAAAGGTGGAGGAATGGCT	59.0	166	NM_204305.2
*Claudin 3*	CCAAGATCACCATCGTCTCC	CACCAGCGGGTTGTAGAAAT	58.0	113	NM_204202.2
*ZO-1*	TCAGACAAAGTTCCCTGCCT	TGGCTAGTTTCTCTCGTGCA	58.9	110	XM_040680632.1
*Occludin*	TCCTCATCGTCATCCTGCTC	TTCTTCACCCACTCCTCCAC	59.0	145	XM_025144247.2
*Jam-2*	AGCCTCAAATGGGATTGGATT	CATCAACTTGCATTCGCTTCA	57.7	59	NM_001397141.1
*Mucin 2*	TGTGTTTGAGAAGTGCCGTG	AGAGCAGCAAACACCATTGG	59.0	184	XM_040673077.1
*TNF-α*	TATCCTCACCCCTACCCTGT	AACTGGGCGGTCATAGAACA	58.8	162	NM_204267.2
*IL-8*	TCCTGGTTTCAGCTGCTCTGT	CGCAGCTCATTCCCCATCT	60.8	61	NM_205498.2
*IFN-γ*	GTAGCTGACGGTGGACCTAT	ATGTGTTTGATGTGCGGCTT	58.8	146	NM_205149.2
*TLR2*	TGCAGTCCAACCAAATCAGC	CGAAGGTGTTGGAGCGAAAA	59.0	142	NM_001396826.1
*TLR4*	GGCACCTACCCTGTCTTTCT	GAGTTGCCTGCCATCTTCAG	59.0	134	NM_001030693.2
*MyD88*	TGCAAGACCATGAAGAACGA	TCACGGCAGCAAGAGAGATT	58.4	123	NM_001030962.5

### Relative Abundance of sIgA in Ileal Mucosa

Ileal mucosa was homogenized with 0.1 M PBS, and the supernatant was centrifuged at 3,000 rpm for 15 min. BCA protein quantitative kit (HuaXingBio, Beijing, China) was used to determine the total protein content of the ileal mucosa homogenate according to the manufacturer's instructions. Concentration of sIgA was determined by an enzyme-linked immunosorbent assay (Nanjing Jiancheng Bioengineering Institute, Nanjing, Jiangsu, China) according to the manufacturer's instructions. Data were acquired using a Microplate reader (SpectraMax M3, Molecular Devices, San Jose, CA, USA) equipped with SoftMax Pro Software. Relative abundance of sIgA in ileal mucosa was expressed as the ratio of sIgA to the total protein concentration of ileal mucosa homogenate.

### Quantification of Cecal Microflora by qRT-PCR

Microbial DNA was isolated from ileal and cecal contents by the stool DNA kit (TIANGEN Biotech, Beijing, China) according to the manufacturer's instructions and was stored at −20°C. Numbers of total bacteria, *Lactobacillus*, and *Escherichia coli* were quantified using real-time PCR in a StepOnePlus system. Each reaction was run in a 10 μl volume containing 5 μl HiPer SYBR Premix EsTaq (MF787-01, Mei5bio, Beijing), 0.3 μl of forward and 0.3 μl of reverse primers (100 nM), 1 μl template DNA, and 3.4 μl of double-distilled water for detecting total bacteria. The PCR profile was processed as follows: one cycle of pre-incubation at 94°C for 180 s; 40 cycles of denaturation at 94°C for 30 s, annealing at 62°C for 30 s, and extension at 72°C for 60 s; one cycle of melting at 95°C for 5 s and 60°C for 60 s; and one cycle of cooling at 50°C for 30 s. To detect *Lactobacillus* and *E. coli*, each reaction was completed in a 10 μl volume containing 5 μl FastFire qPCR PreMix (Probe) (TIANGEN Biotech, Beijing, China), 0.25 μl of forward and 0.25 μl of reverse primers (100 nM), 0.15 μl probes, 1.5 μl template DNA, and 2.85 μl of double-distilled water. The PCR profile was determined as follows: one cycle of pre-incubation at 95°C for 600 s and 40 cycles of denaturation at 95°C for 15 s and annealing at 60°C for 60 s. All samples were analyzed in duplicate. Briefly, primers and fluorescent oligonucleotide probes (Table 3) were obtained from previous studies (26, 27) and commercially synthesized by Beijing Chenhuida Biotechnology Co.

**Table 3:** Sequences of the primer and probe for detection specific for intestinal microflora of broiler.

**Item**	**Prime/probe name and sequence**	**Reference**
Total bacteria	Forward: ACTCCTACGGGAGGCAGCAG Reverse: ATTACCGCGGCTGCTGG	Fierer et al. (26)
*Lactobacillus*	Forward: GAGGCAGCAGTAGGGAATCTTC Reverse: CAACAGTTACTCTGACACCCGTTCTTC Probe: AAGAAGGGTTTCGGCTCGTAAAACTCTGTT	Chen et al. (27)
*Escherichia coli*	Forward: CATGCCGCGTGTATGAAGAA Reverse: CGGGTAACGTCAATGAGCAAA Probe: AGGTATTAACTTTACTCCCTTCCTC	Chen et al. (27)

For the quantification of bacteria in the test samples, specific standard curves were drawn by constructing standard plasmids as described by Han et al. (28). Briefly, the standard strains of *Lactobacillus* (ATCC33323) and *E. coli* (V99 CGMCC1, 12881) were cultured anaerobically or aerobically in respective cultures including 1% glucose at 37°C from 12 to 48 h. The 16S rDNA genes of *Lactobacillus* and *E. coli* were amplified using PCR. Then, the specific PCR product was purified using a Gel Extraction Kit (Beijing ComWin Biotech Co., Ltd., Beijing, China) and cloned into pEASY-Blunt vector (TransGen Biotech, Beijing, China). After verification of the sequence, the recombinant plasmid was isolated using the TIANprep Mini Plasmid Kit (TIANGEN Biotech, Beijing, China). The standard plasmids of *Lactobacillus* and *E. coli* were constructed successfully. The copy numbers of bacteria were calculated using the following formula: (6.0233 ^*^ 10^23^ copies/mol ^*^ DNA concentration (μg/μl))/(660 ^*^ 10^6^^*^ DNA size (bp)). Bacterial counts were performed using serial dilutions (10:1 to 10:8 dilutions) of plasmid DNA to generate the standard curve for total bacteria, *Lactobacillus* and *E. coli*. Each standard curve was constructed by linear regression of mean cycle threshold values against the logarithm of template copy numbers, ranging from 3 to 9 log_10_ copies. Target copy number of each sample was calculated from the standard curve (29, 30).

### Ion Chromatographic Assays of Short-Chain Fatty Acids (SCFAs) and Lactic Acid

Samples of ileal and cecal contents were thawed and thoroughly mixed immediately before testing. Concentrations of acetic, propionic, butyric and valeric, and lactic acids and total SCFAs were determined with a Dionex ICS-3000 Ion Chromatography System as described by Tong et al. (29).

### Data Analysis

Data were analyzed using the ANOVA method in SPSS 20.0, and all data were checked for normal distribution and homogeneous variance. Each pen was regarded as an experimental unit for measurement of growth performance. Each euthanized broiler was regarded as an experimental unit for measurement of other indicators. Different experimental units were independent from each other. Treatment means were separated using Duncan's multiple comparison test. Data in tables are reported as means and pooled standard errors. Data in figures are reported as means ± SEM. Linear and quadratic comparisons were applied to determine the dose effect of Microcin C7 in broilers using orthogonal contrasts in SPSS 20.0. Significant differences were declared when *P* < 0.05, and statistical trends declared when 0.05 < *P* < 0.10.

## Results

### Growth Performance

Broilers fed 6 mg/kg Microcin C7 tended to consume more feed in the starter period (*P* = 0.07; Table 4) and grow faster over the entire supplemental period (*P* = 0.09; Table 4) than the antibiotic groups. The F/G ratio of broilers fed 4 mg/kg Microcin C7 over the entire supplemental period was decreased compared to that of the control group (*P* = 0.03; Table 4). The F/G ratio of broilers fed antibiotics over the entire supplemental period was also significantly decreased compared with that of the control group (*P* = 0.03; Table 4). A linearly higher ADG was observed in broilers fed Microcin C7 during the finisher supplemental period (*P* = 0.05; Table 4). A quadratically lower F/G ratio was observed in broilers fed Microcin C7 during the entire supplemental period (*P* = 0.02; Table 4). A quadratically lower ADFI was observed in broilers fed Microcin C7 during the finisher supplemental period (*P* = 0.04; Table 4). A linearly increased trend of ADFI (*P* = 0.08, Table 4) was observed in broilers fed Microcin C7 during the finisher supplemental period.

**Table 4:** Effects of Microcin C7 on broiler performance^1^,^2^.

**Item**	**Antibiotic**	**Control**	**Microcin C7 (mg/kg)**			**SEM**	***P*** **-value**		
			**2**	**4**	**6**		**ANOVA**	**Linear**	**Quadratic**
**Day 1–Day 21**									
ADFI (g/day)	46.4	49.1	51.2	49.9	50.8	0.59	0.07	0.42	0.54
ADG (g/day)	34.6	37.0	38.4	38.5	37.4	0.58	0.20	0.78	0.25
F/G^3^	1.35	1.33	1.33	1.30	1.36	0.01	0.59	0.68	0.25
**Day 22–Day 42**									
ADFI (g/day)	140.0	147.6	139.2	143.4	152.9	1.89	0.11	0.28	0.04
ADG (g/day)	79.3	80.2	77.4	82.1	85.8	1.10	0.14	0.05	0.19
F/G^3^	1.77	1.84	1.80	1.75	1.79	0.01	0.32	0.24	0.21
**Day 1–Day 42**									
ADFI (g/day)	94.3	99.6	96.3	97.8	103.1	0.65	0.14	0.26	0.08
ADG (g/day)	57.5	59.1	58.4	60.8	62.2	1.06	0.09	0.10	0.48
F/G^3^	1.64^a^	1.70^b^	1.65^ab^	1.61^a^	1.66^ab^	0.01	0.03	0.08	0.02

1 *Each mean represents 6 replications of 10 broilers*.

2 *Antibiotic control = broilers fed a basal diet with 45 mg/kg aureomycin plus 30 mg/kg bacitracin methylene disalicylate. Control = broilers fed a basal diet. Microcin C7 = broilers fed a basal diet containing 2, 4, or 6 mg/kg Microcin C7*.

3 *F/G = feed-to-gain ratio*.

a,b *Means in the same row with different superscripts differ (P < 0.05)*.

### Cytokine and Immunoglobulin Concentrations in Serum

**Figure 1:**
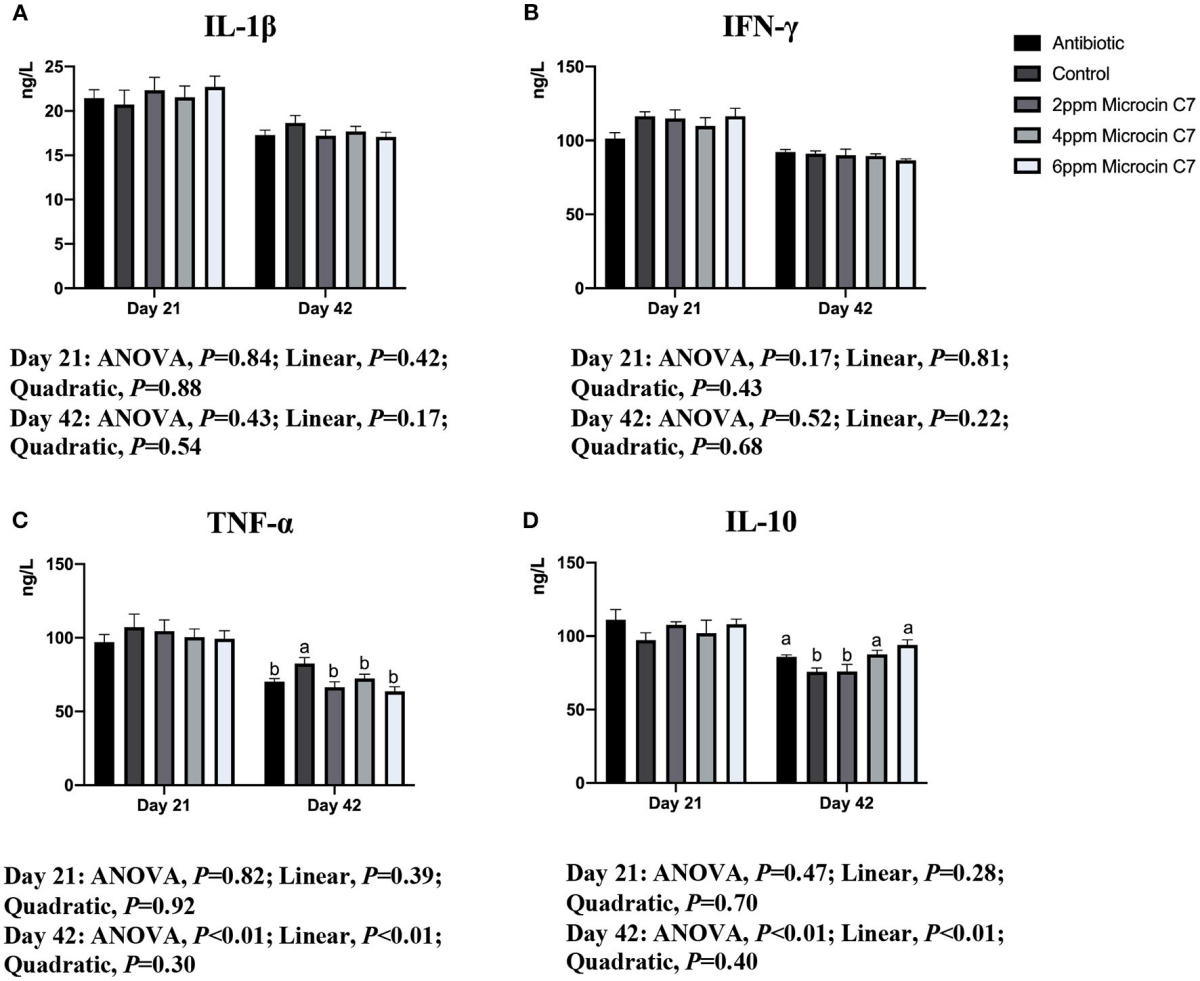
Effect of Microcin C7 on concentration of serum cytokines of broilers. **(A)** Concentration of pro-inflammatory cytokine IL-1β. **(B)** Concentration of pro-inflammatory cytokine IFN-γ. **(C)** Concentration of pro-inflammatory cytokine TNF-α. **(D)** Anti-inflammatory cytokine IL-10 on days 21 and 42. Within the same day, treatments that are significantly different from each other are indicated by different letters above the bar (*P* < 0.05). Bars represent means ± SEM for six broilers per treatment. Antibiotic control = broilers fed a basal diet with 45 mg/kg aureomycin plus 30 mg/kg bacitracin methylene disalicylate. Control = broilers fed a basal diet. Microcin C7 = broilers fed a basal diet containing 2, 4, or 6 mg/kg Microcin C7.

**Figure 2:**
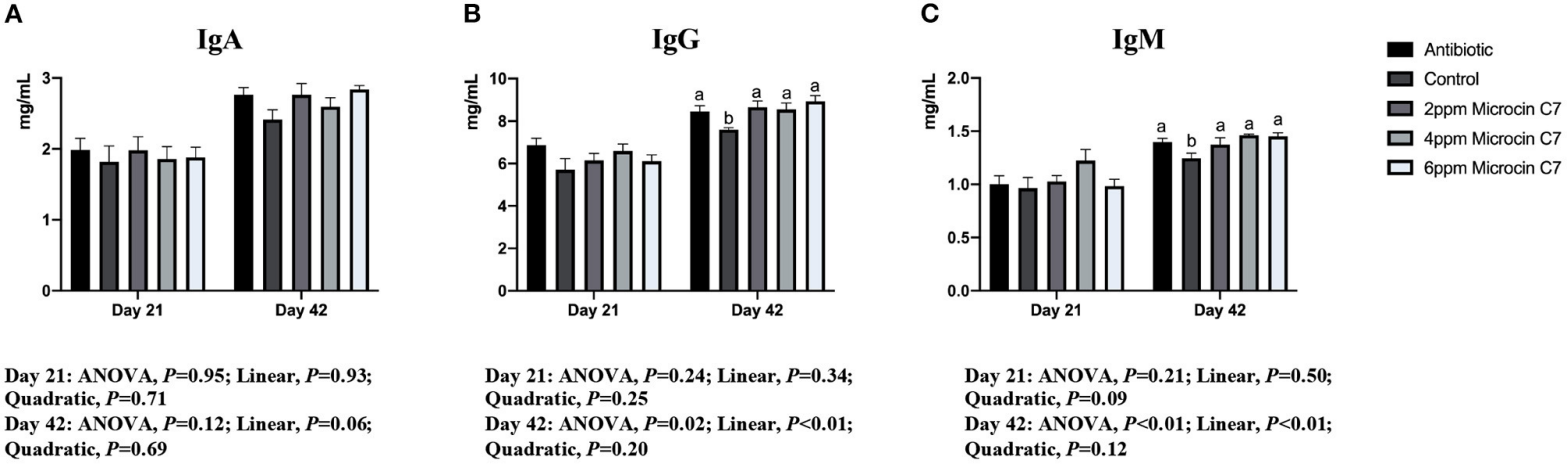
Effect of Microcin C7 on concentration of serum immunoglobulin of broilers. **(A)** Concentration of IgA. **(B)** Concentration of IgG. **(C)** Concentration of IgM. Within the same day, treatments that are significantly different from each other are indicated by different letters above the bar (*P* < 0.05). Bars represent means ± SEM for six broilers per treatment. Antibiotic control = broilers fed a basal diet with 45 mg/kg aureomycin plus 30 mg/kg bacitracin methylene disalicylate. Control = broilers fed a basal diet. Microcin C7 = broilers fed a basal diet containing 2, 4, or 6 mg/kg Microcin C7.

On Day 42, broilers fed with antibiotic, 4 and 6 mg/kg Microcin C7, had increased concentration of the anti-inflammatory cytokine IL-10 (Figure 1) and IgG and IgM (Figure 2) compared with the control group (*P* < 0.05). Broilers fed with antibiotic and Microcin C7 had decreased concentration of pro-inflammatory cytokine TNF-α (Figure 1) compared with the control group (*P* < 0.05). However, all groups were similar with each other on Day 21 (*P* > 0.05). On Day 42, a linearly higher content of IL-10 (Figure 1) and IgG and IgM (Figure 2) was observed in broilers fed Microcin C7 (*P* < 0.05). A linearly lower content of TNF-α was observed in broilers fed Microcin C7 (*P* < 0.05, Figure 1).

### Small Intestinal Morphology

On Day 21, dietary addition of 2, 4, and 6 mg/kg Microcin C7 increased the V/C ratio of ileum and significantly increased the V/C ratio of duodenum and the villus height of jejunum compared with the control group (*P* < 0.05; Table 5). Dietary addition of 2 and 6 mg/kg Microcin C7 increased the villus height of the duodenum compared with the control group (*P* < 0.05; Table 5). Dietary addition of 4 and 6 mg/kg Microcin C7 increased the V/C ratio of jejunum compared with the control group (*P* < 0.05; Table 5). Dietary addition of 2 and 4 mg/kg Microcin C7 decreased the crypt depth of the duodenum compared with the control group (*P* < 0.05; Table 5). Dietary addition of 4 and 6 mg/kg Microcin C7 decreased crypt depth of ileum compared with the control group (*P* < 0.05; Table 5). However, on Day 21, dietary addition of antibiotic decreased villus height and villus height/crypt depth ratio in the duodenum and increased crypt depth and decreased the villus height/crypt depth ratio in the ileum (*P* < 0.05; Table 5). A linearly higher villus height and V/C ratio was observed in the small intestine of broilers fed Microcin C7 (*P* < 0.05; Table 5), and a linearly lower crypt depth was observed in the ileum of broilers fed Microcin C7 (*P* < 0.05; Table 5).

**Table 5:** Effect of Microcin C7 on small intestinal morphology in broilers^1^,^2^.

**Item**	**Antibiotic**	**Control**	**Microcin C7 (mg/kg)**			**SEM**	***P*** **-value**		
			**2**	**4**	**6**		**ANOVA**	**Linear**	**Quadratic**
**Day 21**									
**Duodenum**									
Villus height (μm)	1,179.01^d^	1,269.09^c^	1,331.25^b^	1,309.65^bc^	1,436.74^a^	16.79	<0.01	<0.01	0.06
Crypt depth (μm)	154.81^a^	158.48^a^	145.86^bc^	141.07^c^	150.98^ab^	1.67	<0.01	0.06	0.01
V/C^3^	7.56^d^	8.28^c^	8.92^b^	9.23^b^	9.82^a^	0.15	<0.01	<0.01	0.85
**Jejunum**									
Villus height (μm)	818.24^d^	838.31^d^	895.03^c^	952.13^b^	1,006.26^a^	13.84	0.01	<0.01	0.92
Crypt depth (μm)	132.25	125.39	130.31	132.81	127.54	1.14	0.30	0.62	0.09
V/C^3^	6.33^c^	6.54^c^	6.35^c^	7.00^b^	8.33^a^	0.15	<0.01	<0.01	<0.01
**Ileum**									
Villus height (μm)	632.35^c^	639.98^c^	671.23^b^	740.16^a^	740.70^a^	9.26	<0.01	<0.01	0.05
Crypt depth (μm)	138.84^a^	132.59^b^	133.29^b^	123.69^c^	125.36^c^	1.26	<0.01	<0.01	0.78
V/C^3^	4.44^d^	4.80^c^	4.49^d^	6.21^a^	5.93^b^	0.14	<0.01	<0.01	0.79
**Day 42**									
**Duodenum**									
Villus height (μm)	1,311.71^b^	1,194.74^c^	1,421.70^a^	1,430.85^a^	1,436.99^a^	18.95	<0.01	<0.01	<0.01
Crypt depth (μm)	187.20	181.69	195.69	189.54	171.07	2.84	0.06	0.03	<0.01
V/C^3^	6.97^c^	6.48^c^	6.80^bc^	7.39^b^	8.95^a^	0.18	<0.01	<0.01	<0.01
**Jejunum**									
Villus height (μm)	1,070.46^c^	1,122.06^b^	1,146.67^b^	1,157.40^b^	1,222.91^a^	11.77	<0.01	<0.01	0.24
Crypt depth (μm)	225.76^a^	233.86^a^	221.74^a^	205.34^b^	186.61^c^	3.79	<0.01	<0.01	0.54
V/C^3^	4.73^d^	4.80^d^	5.18^c^	6.06^b^	6.45^a^	0.14	<0.01	<0.01	0.88
**Ileum**									
Villus height (μm)	760.39^b^	717.15^c^	771.81^b^	786.91^b^	820.06^a^	8.78	<0.01	<0.01	0.46
Crypt depth (μm)	137.86^c^	185.39^a^	150.97^b^	154.47^b^	136.25^c^	3.74	<0.01	<0.01	0.05
V/C^3^	5.37^b^	3.96^c^	4.82^b^	5.06^b^	6.12^a^	0.16	<0.01	<0.01	0.60

1 *Each mean represents six broilers*.

2 *Antibiotic control = broilers fed a basal diet with 45 mg/kg aureomycin plus 30 mg/kg bacitracin methylene disalicylate. Control = broilers fed a basal diet. Microcin C7 = broilers fed a basal diet containing 2, 4, or 6 mg/kg Microcin C7*.

3 *V/C = the ratio of villus height to crypt depth*.

a,b,c,d,e *Means in the same row with different superscripts differ (P < 0.05)*.

On Day 42, dietary addition of 2, 4, and 6 mg/kg Microcin C7 increased the villus height of the duodenum and ileum and the V/C ratio of the jejunum and ileum compared with the control group (*P* < 0.05; Table 5). Dietary addition of 4 and 6 mg/kg Microcin C7 increased the V/C ratio of duodenum compared with the control group (*P* < 0.05; Table 5). Dietary addition of 6 mg/kg Microcin C7 increased the villus height of the jejunum compared with the control group (*P* < 0.05; Table 5). Dietary addition of 4 and 6 mg/kg Microcin C7 decreased the crypt depth of the jejunum compared with the control group (*P* < 0.05; Table 5). Dietary addition of 2, 4, and 6 mg/kg Microcin C7 decreased the crypt depth of the ileum compared with the control group (*P* < 0.05; Table 5). On Day 42, dietary addition of antibiotic increased the villus height of the duodenum and villus height and V/C ratio of the ileum and decreased the crypt depth of the ileum compared with the control group (*P* < 0.05; Table 5). However, dietary addition of antibiotic decreased the villus height of the jejunum compared with the control group (*P* < 0.05; Table 5). A linearly higher villus height and a higher V/C ratio were observed in the small intestine of broilers fed Microcin C7 (*P* < 0.05; Table 5), and a linearly lower crypt depth was observed in the duodenum (*P* = 0.03; Table 5), jejunum (*P* < 0.05; Table 5), and ileum (*P* < 0.05; Table 5) of broilers fed Microcin C7.

There was no significant difference in intestinal morphology between all groups (Supplementary Figure S1).

### Gene Expression in Jejunal Mucosa

**Figure 3:**
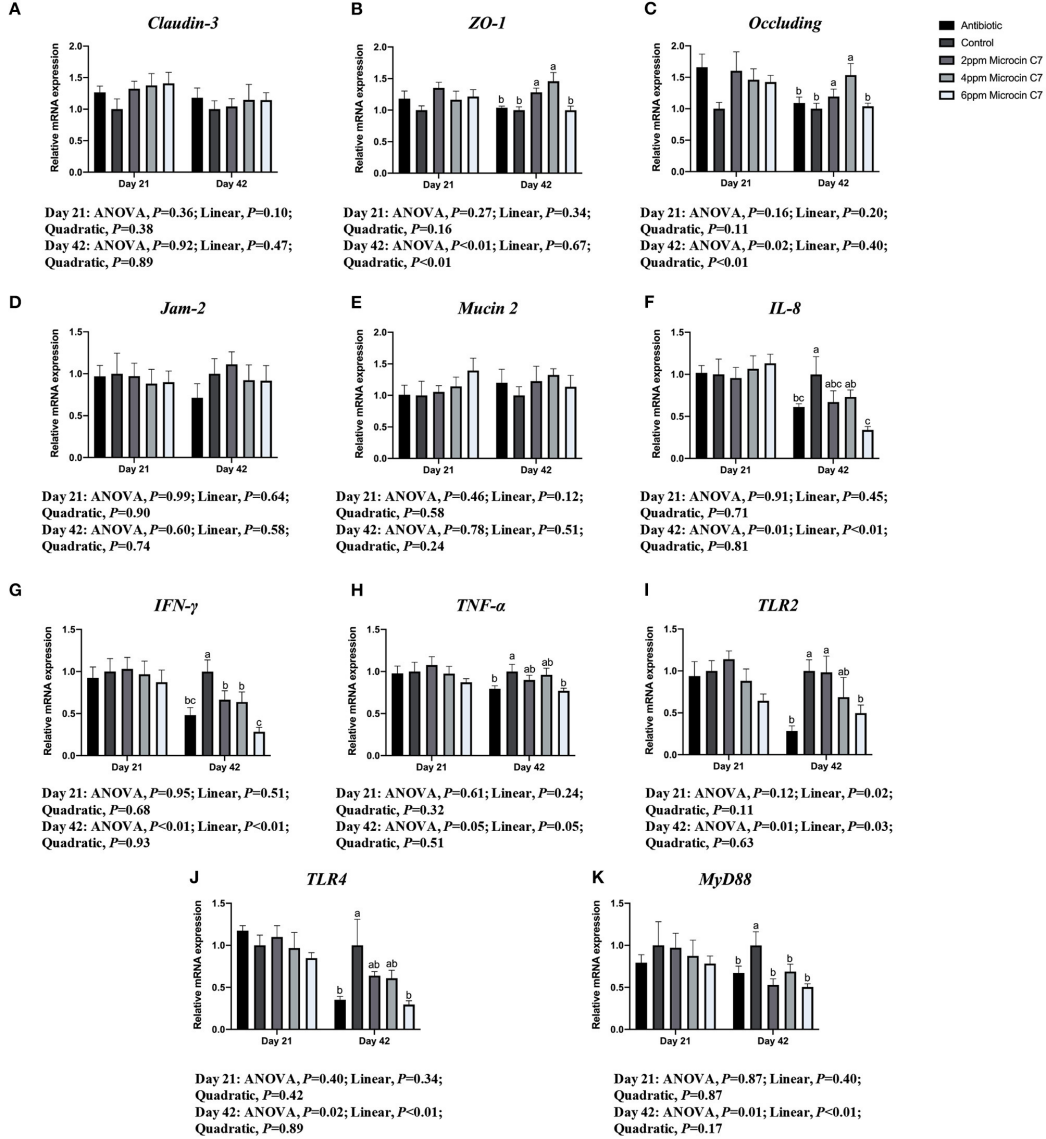
Effect of Microcin C7 on gene expression in the jejunal mucosa of broilers. **(A)** Relative mRNA expression of *Claudin-3*. **(B)** Relative mRNA expression of *ZO-1*. **(C)** Relative mRNA expression of *Occludin*. **(D)** Relative mRNA expression of *Jam-2*. **(E)** Relative mRNA expression of *Mucin 2*. **(F)** Relative mRNA expression of *IL-8*. **(G)** Relative mRNA expression of *IFN-*γ. **(H)** Relative mRNA expression of *TNF-*α. **(I)** Relative mRNA expression of *TLR2*. **(J)** Relative mRNA expression of *TLR4*. **(K)** Relative mRNA expression of *MyD88*. Within the same day, treatments that are significantly different from each other are indicated by different letters above the bar (*P* < 0.05). Bars represent means ± SEM for six broilers per treatment. Antibiotic control = broilers fed a basal diet with 45 mg/kg aureomycin plus 30 mg/kg bacitracin methylene disalicylate. Control = broilers fed a basal diet. Microcin C7 = broilers fed a basal diet containing 2, 4, or 6 mg/kg Microcin C7.

On Day 42, diets supplemented with 4 mg/kg Microcin C7 significantly upregulated expression of *Occludin* compared with the control group (*P* < 0.05; Figure 3). Diets supplemented with 2 and 4 mg/kg Microcin C7 upregulated the expression of *ZO-1* compared with the control group (*P* < 0.05; Figure 3). However, diets supplemented with Microcin C7 had no significant effect on the expression of *Claudin 3, Jam-2*, or *Mucin 2* compared with control group. A quadratically higher gene expression of *Occludin* and *ZO-1* was observed in broilers fed Microcin C7 (*P* < 0.05; Figure 3).

On Day 42, diets supplemented with 6 mg/kg Microcin C7 or antibiotics significantly downregulated the expression of *TNF-*α and *IL-8* compared with the control group (*P* < 0.05; Figure 3). Diets supplemented with Microcin C7 or antibiotics significantly downregulated expression of *IFN-*γ compared with the control group (*P* < 0.05; Figure 3). A linearly lower gene expression of *TNF-*α (*P* = 0.05), *IL-8* (*P* < 0.05), and *IFN-*γ (*P* < 0.05) was observed in broilers fed Microcin C7 (Figure 3).

On Day 42, diets supplemented with 6 mg/kg Microcin C7 or antibiotics significantly downregulated expression of *TLR4* and *TLR2* compared with the control group (*P* < 0.05; Figure 3). Diets supplemented with Microcin C7 or antibiotics significantly downregulated *MyD88* compared with the control group (*P* < 0.05; Figure 3). A linearly lower gene expression of *TLR4* (*P* < 0.05), *TLR2* (*P* = 0.03), and *MyD88* (*P* < 0.05) was observed in broilers fed Microcin C7 (Figure 3).

However, neither Microcin C7 nor antibiotics had any significant effects on expression of tight junction proteins, adhesion junction proteins, pro-inflammatory cytokines, chemokines, pattern recognition receptor, and *MyD88* on Day 21 compared with the control group (*P* > 0.05; Figure 3). However, a linearly lower gene expression of *TLR2* was observed in broilers fed Microcin C7 on Day 21 (*P* < 0.05; Figure 3).

### Total sIgA in Ileal Mucosa

**Figure 4:**
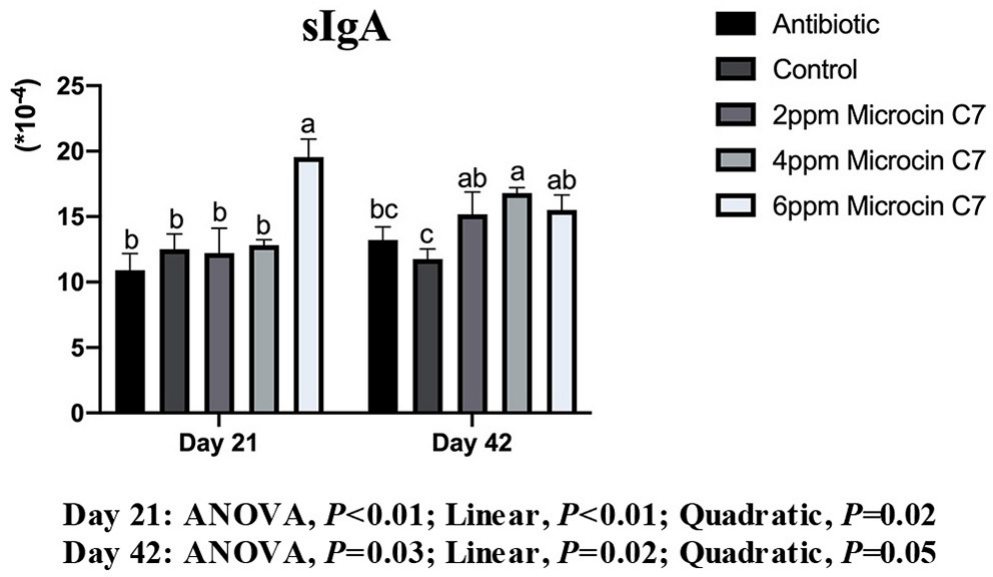
Effect of Microcin C7 on relative abundance of sIgA in the ileal mucosa of broilers. Relative abundance of sIgA in the ileal mucosa was expressed as the ratio of sIgA to the total protein concentration of ileal mucosa homogenate. Within the same day, treatments that are significantly different from each other are indicated by different letters above the bar (*P* < 0.05). Bars represent means ± SEM for six broilers per treatment. Antibiotic control = broilers fed a basal diet with 45 mg/kg aureomycin plus 30 mg/kg bacitracin methylene disalicylate. Control = broilers fed a basal diet. Microcin C7 = broilers fed a basal diet containing 2, 4, or 6 mg/kg Microcin C7.

On Day 21, broilers fed with 6 mg/kg Microcin C7 had significantly increased sIgA concentration compared with the control group (*P* < 0.05; Figure 4). A linearly higher content of sIgA was observed in broilers fed Microcin C7 (*P* < 0.01; Figure 4). On Day 42, broilers fed with Microcin C7 had significantly increased sIgA compared with the control group (*P* < 0.05). A linearly higher content of sIgA was observed in broilers fed Microcin C7 (*P* = 0.02; Figure 4). However, feeding antibiotics to broilers had no significant effect on sIgA concentration (*P* > 0.05).

### Quantification of Cecal Bacteria

**Figure 5:**
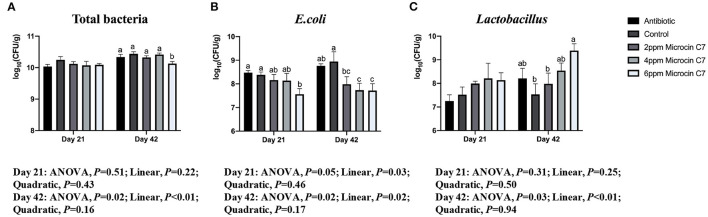
Effect of Microcin C7 on cecal total bacteria, *E. coli* and *Lactobacillus* populations in ceca of broilers. **(A)** Number [log_10_(CFU/g)] of total bacteria. **(B)** Number [log_10_(CFU/g)] of *E. coli*. **(C)** Number [log_10_(CFU/g)] of *Lactobacillus*. Within the same day, treatments that are significantly different from each other are indicated by different letters above the bar (*P* < 0.05). Bars represent means ± SEM for 6 broilers per treatment. Bacterial number is expressed as log10 CFU per gram of wet cecal digesta. Antibiotic control = broilers fed a basal diet with 45 mg/kg aureomycin plus 30 mg/kg bacitracin methylene disalicylate. Control = broilers fed a basal diet. Microcin C7 = broilers fed a basal diet containing 2, 4, or 6 mg/kg Microcin C7.

On Day 21, diet supplemented with 6 mg/kg Microcin C7 decreased the population of *E. coli* compared with the control group (*P* < 0.05; Figure 5). A linearly lower content of the number of *E. coli* (log_10_CFU) was observed in broilers fed Microcin C7 (*P* < 0.01; Figure 5). However, there was no significant difference in the number of total bacteria and *Lactobacillus* compared with controls. On Day 42, diets supplemented with 6 mg/kg Microcin C7 decreased the population of total bacteria (*P* < 0.05; Figure 5) and increased the population of *Lactobacillus* compared with the control group (*P* < 0.05; Figure 5). Diets supplemented with Microcin C7, regardless of concentration, decreased the population of *E. coli* (*P* < 0.05; Figure 5). A linearly lower content of the number of total bacteria (log_10_CFU; *P* < 0.01) and *E. coli* (log_10_CFU; *P* = 0.02) was observed in broilers fed Microcin C7 (*P* < 0.01; Figure 5). A linearly higher content of the number of *Lactobacillus* (log_10_CFU) was observed in broilers fed Microcin C7 (*P* < 0.01; Figure 5).

### Ileal and Cecal SCFAs and Lactic Acid Concentrations

On Day 21, broilers fed with 6 mg/kg Microcin C7 had increased acetic acid, butyric acid, and valeric acids in ileal contents compared with broilers fed the control diet (*P* < 0.05, Table 6). A linearly higher content of acetic acid, butyric acid, and valeric acid was observed in ileal contents of broilers fed Microcin C7 (*P* < 0.05; Table 6). On Day 42, broilers fed 6 mg/kg Microcin C7 had increased lactic acid, acetic acid, and total SCFAs in ileal digesta compared to control broilers (*P* < 0.05, Table 6). A linearly higher content of lactic acid, acetic acid, propionic acid, butyric acid, and total SCFAs was observed in ileal contents of broilers fed Microcin C7 (*P* < 0.05; Table 6).

**Table 6:** Effect of Microcin C7 on lactic acid and SCFA in ileal digesta (mg/kg of wet ileal digesta)^1^,^2^.

**Item**	**Antibiotic**	**Control**	**Microcin C7 (mg/kg)**			**SEM**	***P*** **-value**		
			**2**	**4**	**6**		**ANOVA**	**Linear**	**Quadratic**
**Day 21**									
Lactic acid	903.51	1,097.43	1,315.59	1,323.14	1,377.82	153.01	0.87	0.63	0.84
Acetic acid	233.12^b^	233.47^b^	204.38^b^	222.56^b^	355.58^a^	11.65	<0.01	<0.01	<0.01
Propionic acid	3.23	4.16	3.33	3.69	4.92	0.29	0.35	0.40	0.15
Butyric acid	11.73^a^	9.51^a^	10.18^a^	11.54^a^	15.94^b^	3.78	0.02	<0.01	0.17
Valeric acid	2.67^c^	4.53^b^	4.23^bc^	5.52^b^	7.61^a^	0.38	<0.01	<0.01	0.05
Total SCFA^3^	1,159.78	1,398.26	1,532.17	1,545.23	1,726.86	171.25	0.86	0.58	0.95
**Day 42**									
Lactic acid	5,842.16^ab^	4,579.27^b^	4,857.41^b^	4,924.22^b^	7,138.92^a^	308.88	0.04	0.01	0.13
Acetic acid	318.27^a^	178.22^b^	248.57^ab^	303.04^a^	320.15^a^	14.88	<0.01	<0.01	0.35
Propionic acid	3.29	3.01	3.54	4.85	4.62	0.28	0.13	0.04	0.55
Butyric acid	9.31	6.59	8.10	8.28	9.71	0.39	0.09	<0.01	0.95
Valeric acid	4.64	4.93	6.65	6.32	5.86	0.28	0.08	0.38	0.09
Total SCFA^3^	6,201.98^ab^	4,854.11^b^	5,189.72^b^	5,119.91^b^	7,557.73^a^	312.62	0.02	<0.01	0.09

1 *Each mean represents six broilers*.

2 *Antibiotic control = broilers fed a basal diet with 45 mg/kg aureomycin plus 30 mg/kg bacitracin methylene disalicylate. Control = broilers fed a basal diet. Microcin C7 = broilers fed a basal diet containing 2, 4, or 6 mg/kg Microcin C7*.

3 *Total SCFA contains lactic acid, acetic acid, propionic acid, butyric acid, and valeric acid*.

a,b,c,d,e *Means in the same row with different superscripts differ (P < 0.05)*.

In cecal digesta, the lactic acid, acetic acid, propionic acid, and total SCFAs were higher in broilers fed 6 mg/kg Microcin C7 on Day 21 (*P* < 0.05; Table 7). A linearly higher content of lactic acid, acetic acid (*P* < 0.05; Table 7), propionic acid (*P* = 0.05; Table 7), and total SCFAs (*P* < 0.05; Table 7) were observed in cecal contents of broilers fed Microcin C7. On Day 42, birds fed the highest concentration of Microcin C7 displayed increased lactic acid and propionic acid concentrations compared with control-fed broilers (*P* < 0.05; Table 7). A linearly higher content of lactic acid and propionic acid was observed in cecal contents of broilers fed Microcin C7 (*P* < 0.05; Table 7).

**Table 7:** Effect of Microcin C7 on lactic acid and SCFA in cecal digesta (mg/kg of wet ileal digesta)^1^,^2^.

**Item**	**Antibiotic**	**Control**	**Microcin C7 (mg/kg)**			**SEM**	***P*** **-value**		
			**2**	**4**	**6**		**ANOVA**	**Linear**	**Quadratic**
**Day 21**									
Lactic acid	51.96^c^	88.79^bc^	95.12^b^	121.80^ab^	151.20^a^	8.36	<0.01	<0.01	0.45
Acetic acid	3,016.09^c^	3,282.77^bc^	3,611.57^ab^	3,534.17^ab^	3,961.36^a^	85.77	<0.01	0.02	0.77
Propionic acid	314.87^b^	372.67^b^	367.90^b^	400.76^b^	557.26^a^	23.26	<0.01	0.05	<0.01
Butyric acid	874.78	905.75	933.13	1,008.74	1,026.27	34.37	0.60	0.27	0.96
Valeric acid	79.44	89.99	91.55	95.78	101.48	3.76	0.46	0.35	0.82
Total SCFA^3^	4,410.00^b^	4,767.38^b^	4,916.61^ab^	5,141.41^ab^	5,620.01^a^	128.86	0.03	0.04	0.57
**Day 42**									
Lactic acid	44.69^ab^	33.83^b^	46.11^ab^	56.43^a^	58.33^a^	2.50	<0.01	<0.01	0.24
Acetic acid	4,047.24	3,924.25	3,621.77	3,905.69	3,964.46	72.73	0.44	0.84	0.73
Propionic acid	464.12^b^	567.70^b^	522.88^b^	554.58^b^	780.53^a^	27.10	<0.01	<0.01	<0.01
Butyric acid	1,152.20	1,175.74	1,247.13	1,233.60	1,290.44	40.73	0.85	0.41	0.93
Valeric acid	179.61	160.79	157.29	158.14	168.58	5.00	0.62	0.67	0.58
Total SCFA^3^	6,106.61	5,957.58	5,822.13	5,963.94	6,213.01	99.83	0.79	0.41	0.43

1 *Each mean represents six broilers*.

2 *Antibiotic control = broilers fed a basal diet with 45 mg/kg aureomycin plus 30 mg/kg bacitracin methylene disalicylate. Control = broilers fed a basal diet. Microcin C7 = broilers fed a basal diet containing 2, 4, or 6 mg/kg Microcin C7*.

3 *Total SCFA contains lactic acid, acetic acid, propionic acid, butyric acid, and valeric acid*.

a,b,c,d,e *Means in the same row with different superscripts differ (P < 0.05)*.

## Discussion

Microcin C7 is a ‘Trojan horse’ antimicrobial peptide which can be imported effectively into bacterial cells and inhibits protein synthesis through a mechanism of action that limits development bacterial resistance to drugs (31). In this study, we have demonstrated that broilers fed Microcin C7 tended to express increased ADFI and ADG and a decreased F/G ratio. The positive effect of antimicrobial peptides on growth performance of broilers has been reported previously. Choi et al. reported that diets supplemented with 90 mg/kg of the antimicrobial peptide AMP-A3 improved the overall BW gain of Ross 308 chicks (32). Xie et al. reported that diets supplemented with 100 g/t Partt ABP (mainly composed of plectasin) and 100 g/t full-tide ABP (mainly composed of cecropin) reduced the F/G ratio of commercial 818 broiler chickens during the overall feeding period (33). Hu et al. reported that addition of swine gut intestinal antimicrobial peptides (SGAMPs) improved ADG and reduced the F/G ratio and histological lesions of Arbor Acre broilers under chronic heat stress (34). In the present study, a high dose of Microcin C7 (6 mg/kg) elicited improved growth performance, and an intermediate dose of Microcin C7 (4 mg/kg) supported the lowest F/G ratio, which indicated the beneficial effects of Microcin C7 on the performance of broilers. Broilers fed with antibiotics did not show improved performance except for the F/G ratio during the entire period compared with birds fed control diets, and the effect is not as good as that of Microcin C7.

Apart from their direct antimicrobial activities, antimicrobial peptides also play an important role in innate immunity and serve as regulators of pro- and anti-inflammatory responses (13, 14). Blood biochemistry is an important indicator of animal health. When the body is exposed to pathogens, immune cells produce pro-inflammatory cytokines and chemokines to help eliminate invaders. However, the rapid and excessive expression of pro-inflammatory cytokines such as IL-1β, TNF-α, and IFN-γ, often called a “cytokine storm”, causes cell and organ damage (35, 36). In contrast, anti-inflammatory cytokines such as IL-4 and IL-10 resist the development of inflammatory responses. The balance of pro- and anti-inflammatory cytokines is important for the resolution of inflammatory diseases (37). Wang et al. reported that the antimicrobial peptide Microcin J25 can significantly downregulate pro-inflammatory cytokines, TNF-α, IL-1β, and IL-6, in serum of broilers challenged with *E. coli* or *Salmonella* (38). Yu et al. also reported that diets supplemented with Microcin J25 significantly reduced cytokines IL-1β, IL-6, and TNF-α and increased IL-10 in serum of weanling piglets (36). In the present study, Microcin C7 and the antibiotic diet significantly decreased TNF-α and increased IL-10 in serum on Day 42, which agrees with a previous study (38, 39). This result showed that Microcin C7 has the potential to suppress inflammatory responses and help maintain a desirable balance of immune responses.

Immunoglobulins that have antibiotic activity are secreted by plasma cells after immune stimulation and can directly participate in humoral immunity. Immunoglobulins can neutralize toxins and pathogen infections through specific binding with corresponding antigens, as part of the antigen-clearing activities of B cells (40). Hurtado et al. reported that the human antimicrobial peptide LL-37 increased the sensitivity of human peripheral B cells, enhancing B-cell activation and increasing IgM and IgG production (41). Shan et al. also reported that artificially synthesized antimicrobial peptide lactoferrin effectively increased serum IgA, IgG, and IgM in weanling piglets (42). Similarly, the present study demonstrated that Microcin C7 and dietary antibiotic increased serum IgG and IgM concentrations. This result showed that Microcin C7 has the potential to improve the immune responses elicited by invasion of pathogens.

The small intestine is the primary location for absorption and transport of nutrients (33). Morphological changes reflect the health status of the gut. Increased villus height is related to increased villus absorption surface area, which can enhance nutrient absorption and potentially improve growth performance. Increased villus height also suggests increased epithelial turnover and activation of cell mitosis (34). However, increased crypt depth may reduce secretion of digestive enzymes and nutrient absorption and eventually lower broiler's growth performance (43). Therefore, greater villus height, decreased crypt depth, and greater V/C ratio suggest enhanced ability of the gut to absorb nutrients. Some studies have shown beneficial effects of antimicrobial peptides on intestinal morphology. Wen et al. reported that the antimicrobial peptide CADN had a positive effect on villus height and villus height/crypt depth ratio, but a negative effect on crypt depth of the duodenum and ileum of broilers at 42 days of age (44). Choi et al. reported that broilers fed increasing levels of the antimicrobial peptide-A3 in diets had linearly increased villus height of the duodenum, jejunum, and ileum (32). Fan et al. reported that a mutated rabbit defensin NP-1 improved duodenal morphology by increasing the length of long and thin villi (45). In line with previous studies, the present study shows that Microcin C7 significantly increased villus height, decreased crypt depth, and increased the V/C ratio in the small intestine. The result suggests that Microcin C7 has the potential to maintain a favorable structure of the intestine, promote absorptive capacity, and help maintain gut health.

Intestinal epithelial cells constitute barrier surfaces that separate the host from the external environment. Tight junctions connect adjacent intestinal epithelial cells and regulate intestinal permeability. Tight junctions are composed of junction adhesion molecules, the transmembrane protein Occludin, members of the claudin family, and linker proteins such as the zonula occludens protein family (46, 47). Tight junctions act as a barrier to harmful molecules and provide a pore for the permeation of ions, solutes, and water as appropriate (48). Disruption of the tight junction barrier increases paracellular permeability to luminal antigens, inflammatory factors, and bacterial translocation, which leads to sustained inflammation, tissue damage, and reduced nutrient retention (48, 49). In the present study, we demonstrated that dietary Microcin C7, especially at 4 mg/kg, increased mRNA expression of tight junction proteins, *Occludin* and *ZO-1*, but had no significant effect on *Claudin-3* and *Jam-2*. The ability of 6 mg/kg Microcin C7 to promote gene expression of tight junction protein ZO-1 and Occludin was not as good as 2 and 4 mg/kg Microcin C7. I guess it may due to the high anti-inflammatory properties of 6 mg/kg Microcin C7, which may have an irritant effect on the gastrointestinal tract in the meantime. However, compared with the control group, 6 mg/kg Microcin C7 had no side effect on the mRNA expression of tight junction protein. Identical conclusions were reported by Xie et al. who found that a diet supplemented with 100 g/t Partt ABP (mainly composed of plectasin) and 100 g/t full-tide ABP (mainly composed of cecropin) increased expression of *ZO-1* and *Claudin-3* (33). Feng et al. also reported that antimicrobial peptide Cathelicidin-BF increased the expression level of *ZO-1, Occludin*, and *Claudin-1* in the jejunum and colon of weaned piglets compared with the control group (50). Mucus, which covers the intestinal epithelial surface, is one of the key components of gut barrier integrity (51). Mucin 2 is the main component of the intestinal mucus and acts as a surface cleaner and the first line of immune defense against pathogens (52, 53). Xie et al. reported that a diet supplemented with 100 g/t Partt ABP (mainly composed of plectasin) and 100 g/t full-tide ABP (mainly composed of cecropin) increased expression of *Mucin 2* (33). However, our results reported herein show that Microcin C7 had no significant effect on *Mucin 2*. The difference from Xie et al. may be the result of different types of antimicrobial peptides, broilers, or rearing environments used in two studies.

The gut harbors approximately 80% of the immune cells of the whole body and considered to be the largest immunological organ in the body. Consequently, the gut plays a central role in immune regulation and defense against pathogens and influences the overall health of the body (54). Intestinal epithelial cells sense bacterial invasions and activate the immune system by means of pattern recognition receptors (PRRs), such as Toll-like receptors (TLRs) (55). When pathogens invade, TLR signaling pathways are activated, which promote inflammation and cause inflammatory bowel diseases (IBD). TLR2 senses the presence of bacterial lipoproteins, lipoteichoic acids, peptidoglycan, and zymosan. TLR4 is the predominant receptor for LPS from gram-negative organisms. TLR2 and TLR4 expressions are highly upregulated in IBD patients (56). The activation of TLR signaling transduction pathway is initiated by the LPS-LBP-CD14 complex, which signals through the induction of a key linker molecular MyD88, serine kinase IL-1R-associated kinase 4 (IRAK4), and adaptor protein TNF receptor-associated factor 6 (TRAF6). These molecules lead to activation of the transcription factor NF-κB and activating protein 1 followed by cascades of mitogen-activated protein kinases (57, 58). This triggers the induction of numerous genes including pro-inflammatory cytokines such as *IL-1*β, *IL-6, TNF-*α, *IFN-*γ, chemokines such as *IL-8*, and antigen-presenting molecules. Some researchers showed the immune-regulating ability of antimicrobial peptides. Xie et al. reported that a diet supplemented with 100 g/t Partt ABP (mainly composed of plectasin) and 100 g/t full-tide ABP (mainly composed of cecropin) significantly decreased the expression of pro-inflammatory cytokines *IL-17A* and *IFN-*α, which suppress intestinal inflammation (33). Feng reported that the antimicrobial peptide Cathelicidin-BF suppressed pro-inflammatory cytokines *IL-6* and *IL-22* and the chemokine *IL-8* in the jejunum and ileum (50). Yi et al. reported that the antimicrobial peptide Cathelicidin-WA decreased gene and protein expressions of *TLR4* and *MyD88* in the jejunum (59). In line with previous studies, we demonstrated herein that Microcin C7 suppressed mRNA expression of *TLR-2, TLR4, MyD88*; pro-inflammatory cytokines *TNF-*α and *IFN-*γ; and the chemokine *IL-8*. This result showed that Microcin C7 has the potential to attenuate intestinal inflammation and aid in the regulation of the immune system.

Secretory IgA is the predominant mucosal immunoglobulin in mammals and birds. sIgA provides immunological defense by preventing pathogens from adhering to and penetrating the mucosal epithelium and helping maintain symbiotic relationships with commensal bacteria (60). Classically, sIgA eliminates pathogens by inactivating bacterial enzymes and toxins and blocking bacterial attachment (60, 61). Apart from that, sIgA also plays an important role in induction of tolerance to innocuous food antigens and commensal bacteria under normal conditions (62). Bao et al. reported that administration of a pig antimicrobial peptide enhanced sIgA expression in both the duodenum and jejunum of broilers compared with the control group (61). In another study, researchers showed that antimicrobial peptides derived from rabbit sacculus rotundus increased the area of IgA-secreting cells in various intestinal segments (63). The present study showed that administration of Microcin C7 could enhance sIgA expression in the ileum of broilers. However, the dietary antibiotic had no effect on the expression of sIgA. Antibiotic has a similar effect to Microcin C7. This result showed that in addition to bactericidal ability, Microcin C7 can also strengthen the host's immunity in the intestinal mucosa.

Antimicrobial peptides have broad-spectrum activity against gram-negative and gram-positive bacteria (64, 65), fungi (66), eukaryotic parasites (67), and viruses (68) *in vitro* (69). Previous researchers demonstrated that antimicrobial peptides maintain the equilibrium of intestinal microecology by suppressing harmful pathogens and promoting proliferation of beneficial microorganisms *in vivo*, which improved intestinal health. Wen and He reported that the antimicrobial peptide CADN decreased aerobic bacterial counts in both jejunal and cecal digesta of broilers in a dose-dependent manner (44). Choi et al. reported that broilers fed antimicrobial peptide-A3 had fewer excreta coliforms, total anaerobic bacteria, and *Clostridium* spp. than broilers fed control diets (32). Zhang et al. reported that dietary antimicrobial peptide plectasin significantly decreased the quantity of *E. coli* and increased the ratio between *Lactobacilli* and *E. coli* in the ileal contents of 21-day-old yellow-feathered chickens (16). Tang et al. also reported that the antimicrobial peptide lactoferrin significantly decreased the number of *E. coli* and increased the number of *Lactobacilli* and *Bifidobacterium* in the ileum, caecum, and colon of weaned piglets (70). In agreement with previous studies, our study reported herein showed that Microcin C7 decreased total bacteria and *E. coli* and increased *Lactobacillus* in the cecum of broilers, which indicated that Microcin C7 has the potential to selectively regulate gut microbiota. Antibiotics also can potentially regulate intestinal microbiota, but effects were not as good as Microcin C7.

SCFAs and other organic acids such as lactate and succinate are primary microbial fermented products degraded from dietary carbohydrates through an extensive set of enzymes (71). SCFAs are important fuels for intestinal epithelial cells and regulate their proliferation and differentiation, which affect gut motility. SCFAs reduce the pH of gut lumen, inhibit the growth of harmful bacteria, and strengthen gut barrier function and host metabolism (72). Yu et al. reported that dietary Microcin J25 increased lactate and SCFAs in feces of weanling piglets (39). Yi et al. also reported that the antimicrobial peptide CWA increased SCFA levels in feces of weanling piglets (59). In the current study, we found that Microcin C7 increased lactate and SCFA concentrations in the ileum and cecum of broilers, which indicates that Microcin C7 has the potential to adjust the balance of the intestinal microecology and maintain gut health. *Lactobacillus* is the most abundant genus in the duodenum (36–97%), jejunum (39–72%), and ileum (24–96%) at all ages. In the ileum, the abundances of *Lactobacillus* are stable from 7 to 21 days of age and increased to 96.7% on Day 42 (73). Therefore, in the current study, the content of lactic acid is most abundant in the ileum followed by acetic acid. A sharp increase of lactic acid from Day 21 to Day 42 can be seen in our study. In the cecum, acetic acid, propionic acid, and butyric acid are the most abundant SCFAs, but the content of lactic acid is little. Cecal acetate, propionate, butyrate, and valerate acids increase with age (73). This trend can be also seen in our study. The relatively large intra-group variation of SCFAs may be an important reason to explain the different concentrations of SCFAs between birds at age of 21 and 42 days.

## Conclusions

In conclusion, results obtained in this study indicated that dietary supplementation with Microcin C7 can strengthen immune functions, improve intestinal villus structures, enhance intestinal barrier function, and regulate composition of intestinal microbiota, which suggests its potential to improve growth performance. Microcin C7 fed at 4 mg/kg has the best effect on the F/G ratio and expression of tight junction proteins. Microcin C7 fed at 6 mg/kg has the best effect on growth performance, immune and anti-inflammatory functions, and maintenance of intestinal homeostasis. Microcin C7 appears to have the greatest effects in the finisher stage of broiler production. This may include two reasons. First, the effect of Microcin C7 is cumulative. Second, broilers intake more Microcin C7 during the finisher stage. These results indicated that Microcin C7 can be used as a promising alternative to traditional antibiotics.

As for overall mode of action for Microcin C7 peptide, we suppose that gram-negative pathogens secrete enterotoxin, release lipopolysaccharide, impair the intestinal barrier, and further result in inflammatory bowel disease (48, 49, 56). Microcin C7 exhibits antimicrobial activity against those pathogens, resists pathogen invasion, and regulates gut microbiota. As an immunomodulator, Microcin C7 can also regulate intestinal immune functions and maintain intestinal microecological balance. Since the intestine is the largest immune and absorption organ, the enhancement of intestinal health by Microcin C7 improves the serum index and further improves growth performance. The specific mechanism responsible for these positive observations will be verified by bacterial infection or DSS-induced colitis model in our future studies.

## Data Availability Statement

The raw data supporting the conclusions of this article will be made available by the authors, without undue reservation.

## Ethics Statement

The animal study was reviewed and approved by China Agricultural University Animal Care and Use Committee.

## Author Contributions

ZD: conceptualization, methodology, investigation, formal analysis, data curation, and writing-original draft. LS: investigation, methodology, and writing–review & editing. FW: resources, investigation, and writing–review & editing. XZ: conceptualization, methodology, and writing-review & editing. HY: methodology and writing-review & editing. LL: formal analysis and writing-review & editing. JZ: conceptualization. SQ: conceptualization, resources, supervision, investigation, and writing–review & editing. All authors have read and agreed to the published version of the manuscript.

## Funding

This study was supported by the National Key Research and Development Program of China (grant number 32030105), Chongqing Rongchang Agricultural and Animal Husbandry High-tech Industry Research and Development Project (cstc2019ngzx0019), and Beijing Swine Innovation Team of Modern Agriculture Industry Technological System.

## Conflict of Interest

The authors declare that the research was conducted in the absence of any commercial or financial relationships that could be construed as a potential conflict of interest.

## Publisher's Note

All claims expressed in this article are solely those of the authors and do not necessarily represent those of their affiliated organizations, or those of the publisher, the editors and the reviewers. Any product that may be evaluated in this article, or claim that may be made by its manufacturer, is not guaranteed or endorsed by the publisher.

## Abbreviations

AMPs: antimicrobial peptides
ADG: average daily gain
ADFI: average daily feed intake
F/G: feed/gain
V/C: villus height/crypt depth
PBS: phosphate-buffered solution
IL-1β: interleukin 1β
IL-8: interleukin 8
IL-10: interleukin 10
TNF-α: tumor necrosis factor α
IFN-γ: interferon-γ
TLR2: toll-like receptor 2
TLR4: toll-like receptor 4
IgA: immunoglobulin A
IgG: immunoglobulin G
IgM: immunoglobulin M
sIgA: secretary immunoglobulin A
ZO-1: Zonula Occludens
Jam-2: junctional adhesion molecule 2
MyD88: myeloid differentiation factor 88
SCFAs: short-chain fatty acids
ANOVA: analysis of variance
DSS: dextran sulfate sodium.
