# Supplementary material for: Effects of Antimicrobial Peptide Microcin C7 on Growth Performance, Immune and Intestinal Barrier Functions, and Cecal Microbiota of Broilers
Source: Front Vet Sci. 2022 Jan 7;8:813629. doi: 10.3389/fvets.2021.813629 (PMC8780134; doi:10.3389/fvets.2021.813629)
Supplement: Supplementary file 1 [file Table_1.DOCX]

Supplementary Material

**Figure S1.** Photomicrograph of cross section of duodenum, jejunum, and ileum from broilers on (A) Day 21 and (B) Day42. H-E staining, 40X. Antibiotic control = broilers fed a basal diet with 45ppm Aureomycin plus 30ppm Bacitracin methylene disalicylate. Control = broilers fed a basal diet. Microcin C7 = broilers fed a basal diet containing 2, 4, or 6 ppm Microcin C7.
