# Supplementary figures and images for: Effects of Antimicrobial Peptide Microcin C7 on Growth Performance, Immune and Intestinal Barrier Functions, and Cecal Microbiota of Broilers
Source: Front Vet Sci. 2022 Jan 7;8:813629. doi: 10.3389/fvets.2021.813629 (PMC8780134; doi:10.3389/fvets.2021.813629)

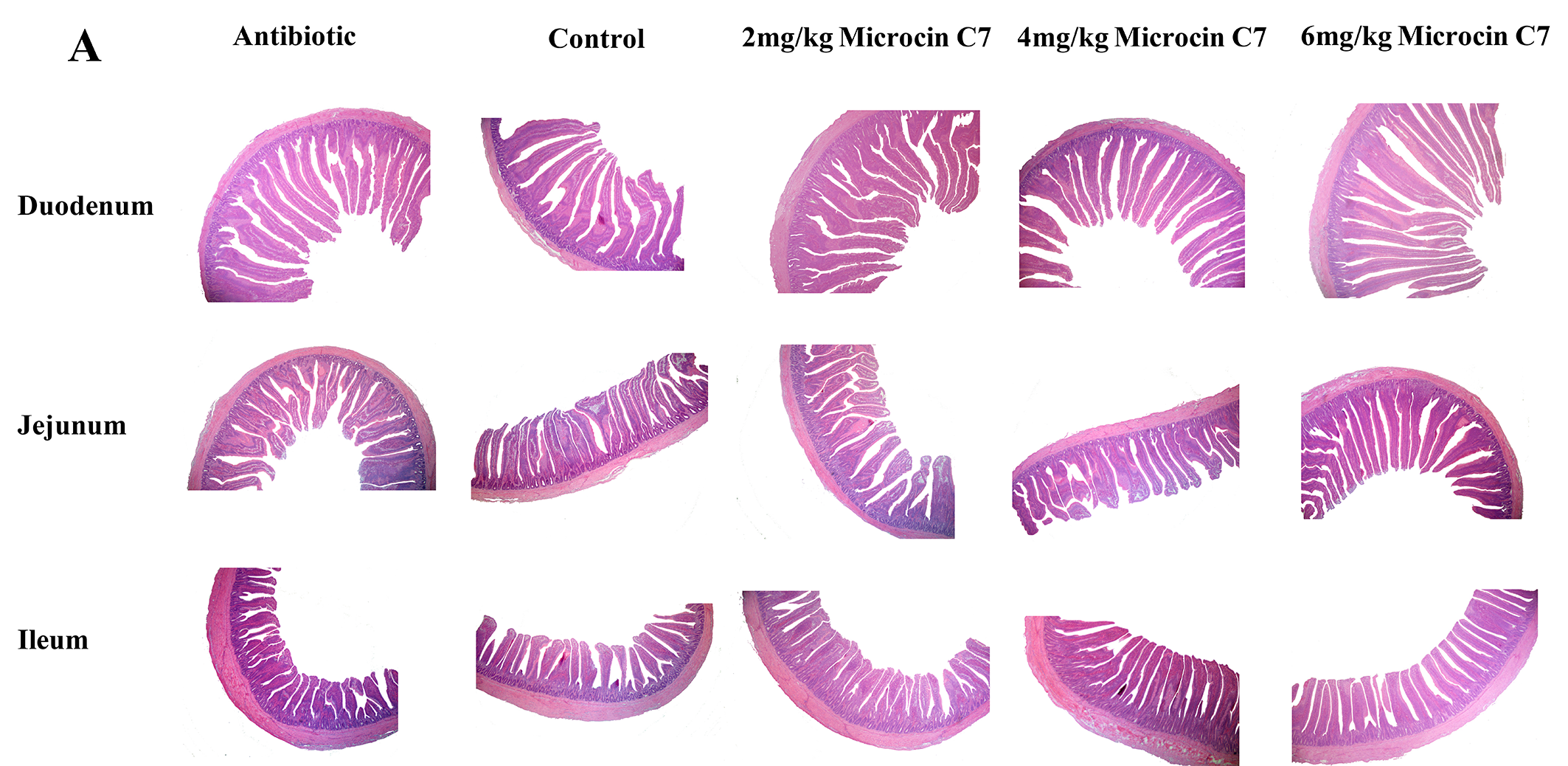

Supplement: Supplementary file 2 [file Image_1.tif]

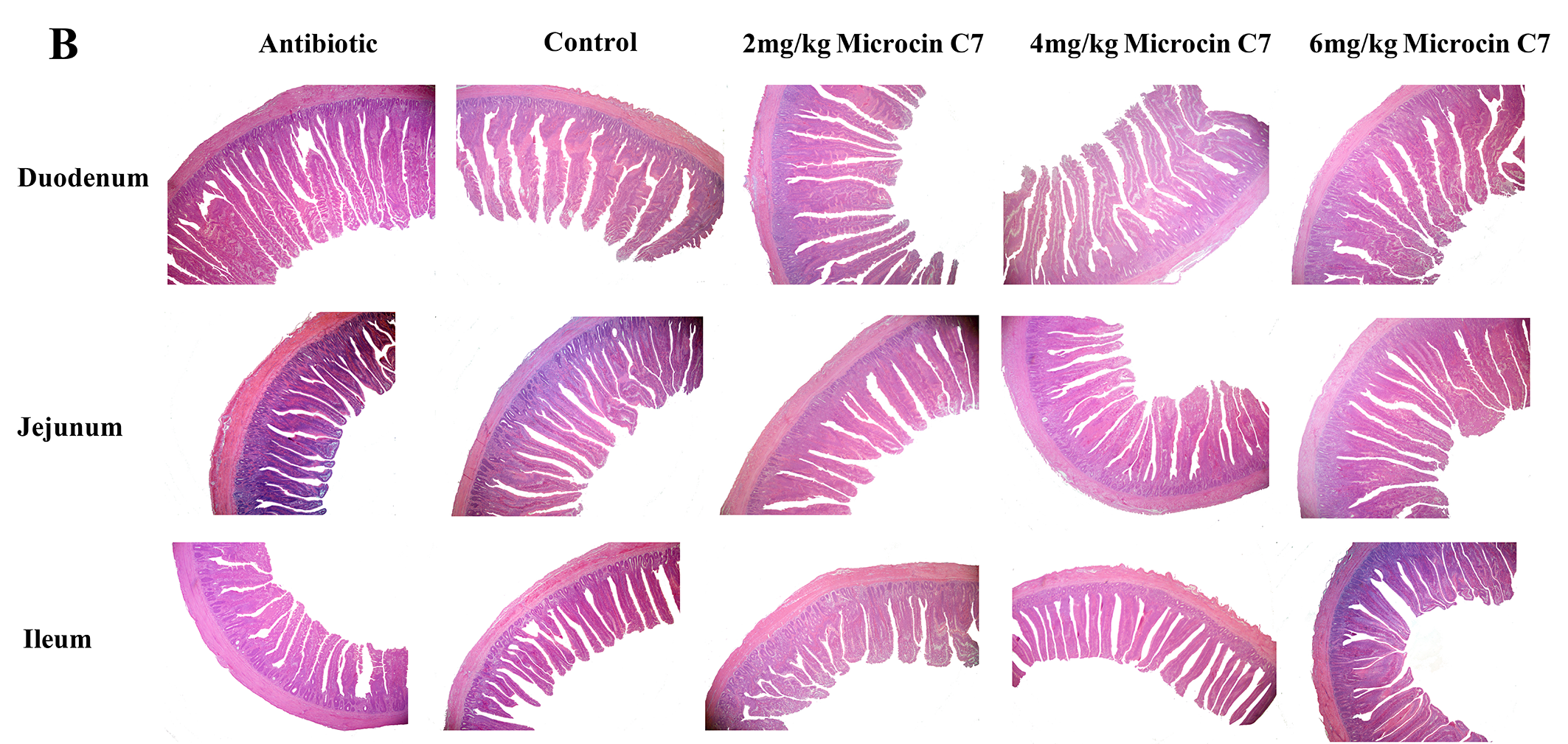

Supplement: Supplementary file 3 [file Image_2.tif]
